# Ultrasonically actuated neural probes for reduced trauma and inflammation in mouse brain

**DOI:** 10.1038/s41378-022-00438-3

**Published:** 2022-11-02

**Authors:** Po-Cheng Chen, Catharine G. Young, Chris B. Schaffer, Amit Lal

**Affiliations:** 1 SonicMEMS Laboratory, School of Electrical and Computer Engineering, Cornell University, Ithaca, NY USA; 2 Meinig School of Biomedical Engineering, Cornell University, Ithaca, NY USA

**Keywords:** Biosensors, Bionanoelectronics

## Abstract

Electrical neural recordings measured using direct electrical interfaces with neural tissue suffer from a short lifespan because the signal strength decreases over time. The inflammatory response to the inserted microprobe can create insulating tissue over the electrical interfaces, reducing the recorded signal below noise levels. One of the factors contributing to this inflammatory response is the tissue damage caused during probe insertion. Here, we explore the use of ultrasonic actuation of the neural probe during insertion to minimize tissue damage in mice. Silicon neural microprobes were designed and fabricated with integrated electrical recording sites and piezoelectric transducers. The microprobes were actuated at ultrasonic frequencies using integrated piezoelectric transducers. The microprobes were inserted into mouse brains under a glass window over the brain surface to image the tissue surrounding the probe using two-photon microscopy. The mechanical force required to penetrate the tissue was reduced by a factor of 2–3 when the microprobe was driven at ultrasonic frequencies. Tissue histology at the probe insertion site showed a reduced area of damage and decreased microglia counts with increasing ultrasonic actuation of the probes. Two-photon imaging of the microprobe over weeks demonstrated stabilization of the inflammatory response. Recording of electrical signals from neurons over time suggests that microprobes inserted using ultrasound have a higher signal-to-noise ratio over an extended time period.

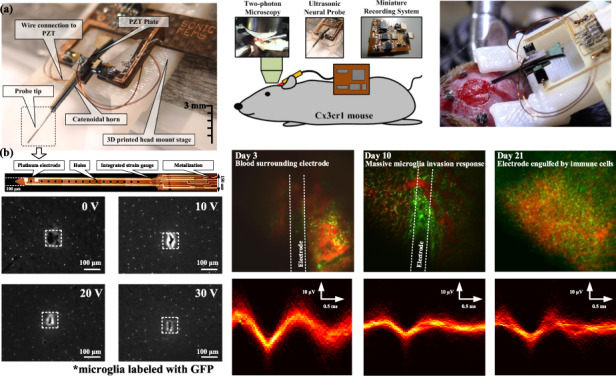

## Introduction

Advanced prosthetics can utilize neural interfaces to record large-scale neuronal activity patterns and use that information to control prosthetic actuators. Hence, a reliable neural interface technology holds promise for treating patients who suffer from limb loss, spinal cord injury, and other neuropathies^1^. By implanting electrodes that can record and stimulate neural activity with a high spatial and temporal resolution, these technologies can use a patient’s cortical activity to control external devices. Although a neural interface’s lifetime can be limited by the neurons themselves being no longer functional in the brain after some time, probe-insertion trauma is considered a very important indicator of useful probe recording lifetime. It is generally accepted^2^ that a significant limitation in achieving a reliable long-term stable neural-probe interface is the brain’s inflammatory response following injury upon insertion.

Neural interfaces can be categorized by the degree of invasiveness (i.e., the extent of tissue damage) and by the degree of spatial resolution of brain tissue recordings. For example, electroencephalography (EEG) monitors voltage fluctuations resulting from ionic currents within the neurons by placing electrodes along the scalp, which causes minimal damage to tissue. However, EEG provides low spatial resolution signals representing millions of neurons. In contrast, electrocorticography (ECoG), in which electrodes are placed directly on the brain’s exposed surface, is more invasive but can achieve better spatial resolution. Microelectrode arrays (MEAs), in which multiple electrodes are implanted into a specific brain location, can obtain the best spatial resolution—enough to resolve individual neuron activity. However, the insertion and continued presence of MEAs typically causes significant damage to the brain tissue and leads to a severe inflammatory response in the brain. This damage and inflammation limit the time over which high-quality recordings can be obtained and risks serious brain tissue injury.

Neurons constitute ~25%^3^ of the tissue in the cerebral cortex, while the rest of the brain matter includes glial cells and vasculature^4^. Glial cells include (1) oligodendrocytes, which create myelin that wraps long-range axons, (2) astrocytes, which support the energy metabolism of neurons and comprise ~30–65%^5^ of the glial cells, and (3) microglia, which are the critical effectors of the brain inflammatory response and makeup ~5% of the glial cells^6^. Microglia play a surveillance role in the uninjured brain and respond rapidly to any adverse stimulus. Both microglia and astrocytes change phenotype when they become activated by an inflammatory response, with astrocytes providing directional cues for the inflammatory reaction through chemical signaling^7^ and microglia migrating toward the injury site and becoming phagocytic and sometimes cytotoxic^8^. Electrode insertion-induced damage drives such inflammation, which leads to an increase in the density of activated microglia near the electrode. These activated microglia may damage nearby neurons, reducing the number of cells available for recording. A glial scar may encapsulate the electrode, reducing the signal-to-noise ratio (SNR) of the recordings to unusable levels^6^. Hence, it is critical to reduce the inflammatory response that occurs in response to neural-probe insertion.

Many researchers have observed a loss of electrode function during long-term recording, with usable recording lifetimes varying from 15 to 81 weeks^7,9,10^. The inflammation that contributes to this degradation is typically thought to be due to the initial insertion trauma over short timescales, and a chronic foreign-body response on longer timescales. The initial insertion of an electrode can directly kill or injure cells, sever capillaries leading to hemorrhage and edema, and cut through the extracellular matrix and cellular processes, all events that trigger microglial activation. This initial inflammatory response to the injury is followed by a chronic foreign-body response where a glial scar forms surrounding the electrode within a few weeks after insertion^11^. By developing quantitative methods to assess tissue-electrode reactions in vivo and ex vivo, we seek to better understand these various stages of response and develop strategies to mitigate them and improve device lifetime.

Several research efforts aim to modify the interactions between the probe and tissue using surface treatments of the probes and recording sites to limit the chronic-phase inflammatory response. For example, ceramic nanostructured coating of electrodes led to better adhesion of astrocytes, leading to a more reliable neural interface^12^. In contrast, a biocompatible inert coating that prevents astrocyte attachment^13^ has also been shown to increase reliability. Other coating approaches have been shown to enhance cortical neuronal attachment^14^, improve neural growth^15^, and decrease electrode impedance (e.g., with poly(3,4-ethylenedioxythiophene) (PEDOT) coating)^16–18^. Another hypothesis to increase the probe recording lifetime is to give the probe an elastic modulus similar to that of the surrounding tissue. With externally induced forces, mechanical perturbations would lead to motion of the probe relative to the tissue. Micromotions during insertion and operation of stiffer probes can cause damage to the brain, while more compliant probes would bend under applied loads with the tissue, reducing microprobe-induced damage. Furthermore, during the probe lifetime, any flexing of the probe due to mechanical moments due to brain motion loading would induce less stress in the tissue, reducing inflammatory reactions. Silicon-based electrodes have a very high elastic modulus mismatch to tissue: 172 GPa for silicon and 0.1 MPa for brain tissue. Therefore, probes have been made from flexible materials such as polyimide and parylene^19–22^. While thinner and more flexible probes generally lead to reduced damage in tissue, they suffer from buckling during insertion if the probe is not sufficiently stiff to cut tissue. While significant effort has been invested in reducing chronic-phase responses that limit the recording lifetime, less work has focused on reducing the initial trauma from insertion or the resulting immediate inflammatory response.

The premise of our work is that reducing the extent of the initial tissue injury and the resulting acute inflammation could serve to limit the overall degree of inflammation over time and thus extend the useful electrode lifetime. This approach could be complimentary to efforts aimed at reducing the chronic foreign-body response. Several attempts have been made to reduce the acute inflammatory response to injury caused during probe insertion. For silicon electrodes, the effect of electrode tip shape, probe size, tip angle, and shaft number has been investigated as a potential way to reduce damage to cells^23–25^. It has been demonstrated that a chisel-tipped silicon electrode shape can produce a damage zone less than 10 µm^26^ wide for a probe cross-section of 40 × 50 µm, while nonsharp probe tips produced more widespread damage. In addition to mechanical properties of probes, electrode insertion velocity may have an effect on tissue mechanical and biological responses. Penetration forces decrease as the probe cross-section decreases; however, there is a trade-off between insertion force and probe stability (i.e., the resistance to buckling)^27^. Hence, probe insertion approaches have been studied to reduce probe-induced damage.

Current neural-probe insertion methodologies are highly dependent on the flexural rigidity of the probe and the number of neural probes in the array. For example, an array of 128 50-µm-diameter microwires was implanted by a hydraulic micropositioner moving at a speed of 1.6 µm/s^28^. Such slow insertion is needed to allow the tissue to relax around each of the relatively flexible probes. On the other hand, the Utah multielectrode array is implanted by a high-speed piston moving at a speed of 8.3 mm/s^29–31^. The probes in the Utah array also have a diameter of 50 µm, but with their short 1.5 mm length provide a high flexural rigidity of 2.8 MNm^2^, enabling insertion into tissue without bending. At the high insertion velocity, the tissue is inertia-stiffened owing to the tissue viscoelastic response leading to decreased tissue deformation, even with a large number of closely spaced electrodes. Michigan-style probes, with lower flexural rigidity, are typically implanted using a linear motor at a speed of 2 mm/s^2^ (see ref. ^32^). While these studies have arrived at insertion parameters that appear to work for scientific measurements, there are likely still opportunities to further improve the insertion mechanics. In this paper, we explore the use of ultrasonic probe tip oscillations to reduce insertion trauma and the resulting inflammatory response.

Mechanically driving a probe at ultrasonic frequencies leads to an oscillating force on the tissue during insertion. The high tip velocity inertia stiffens the tissue, minimizing tissue deformation and reducing velocity-dependent friction between the tissue and the probe. Silicon ultrasonic horn transducers driven by bulk piezoelectric plates vibrating in the longitudinal resonant mode have been shown to produce high vibration amplitudes comparable to traditional titanium alloy^33^ ultrasonic transducers. In this paper, we describe a silicon-based neural probe with recording electrodes that can be vibrated at ultrasonic frequencies during insertion. Our hypothesis was that an ultrasonically actuated neural probe can minimize both mechanical stress and damage during and after insertion. To test this hypothesis, we used postmortem histology, in vivo two-photon imaging, and electrical recording techniques to measure tissue injury, the acute and chronic inflammatory response, and the reliability of neural recording over time.

## Results

### Model of probe insertion under ultrasonic actuation

Ultrasonic-enabled insertion can be distinguished from conventional insertion by the specific motion characteristics of the inserting tool, as the conventional movement of the device is augmented by ultrasonic vibration. In a plunge-type insertion (the main vibration axis and the moving axis of the inserting tool are identical, and the vibration axis is perpendicular to the tissue surface), the stress and strain action on the tissue due to the macroscopic feed motion is intensified or diminished by a periodical stress with a high frequency and a low amplitude. Stress and strain are mainly exerted in the separation zone where the tip is in contact with the tissue. The effective cutting velocity *U* can be expressed as the sum of the linear velocity of the feeding tool, *U*_*dc*_, and the vibration tip velocity, *U*_*ac*_, in the following expression:

1 $$U = U_{dc} + U_{ac} = U_{dc} + \omega A \cdot e^{j\omega t}$$

where *A* is the vibration amplitude, *ω* is the angular frequency, and *t* is time. Ultrasonic insertion can be divided into three different phases: (1) the tip is moving into and cutting the tissue (active phase), (2) the tip is retracting away from the tissue (passive phase), and (3) the tip is approaching the tissue (passive phase). During the active cutting phase, the tip velocity is high enough such that there is no stress relaxation in the tissue. The high tip velocity and the elastic and viscoelastic resistance from the tissue make the tissue stiffer, as it cannot be moved as fast as the tip. This changes the mode of tissue cutting, in the sense of material properties, from ductile cutting to brittle fracture^34^. The force on the probe as it penetrates the tissue includes elastic and viscous forces. The elastic forces are applied when pressing the probe into the uncut part of the tissue. The viscous force term is the force due to the probe moving in the tissue. These reaction forces are balanced by the average ultrasonic cutting force, and the net force applied. If the probe enters at a constant velocity by controlled insertion, the acceleration of the probe can be ignored. The net forces can then be written as

2 $$F_x + F_{US} = C_1 + d \cdot C_2\dot x$$

In Eq. (2), the ultrasonic cutting force can be modeled as being proportional to the transducer drive voltage, *F*_*US*_ = *γV*, where *γ* is a scale factor. This is because the drive voltage controls the probe motion amplitude, which determines the probe tip acceleration that cuts the tissue. The constant term *C*_1_ accounts for the average reaction force from the tissue as the tissue is being pressed by the probe, similar to the Hertzian contact force. In the experiments conducted in this study, a tissue simulant was made of water and agar mixtures, resulting in higher viscosity for higher agar concentrations^35^. The constant *C*_1_ = *C*_10_ + *C*_11_*P*_*A*_ models the elastic force of the tissue, which can be written as a constant in addition to a term that is proportional to the agar concentration. As the probe goes deeper into tissue to a depth *d*, the probe area that is in frictional contact with tissue increases in proportion to *d* and the average insertion velocity *x*˙. The constant *C*_2_ = *βP*_*A*_ models the viscosity of the tissue, which increases with the agar concentration as the number of bonds to be broken with the probe increases. Using these assumptions in Eq. (2), the applied force *F*_*x*_ can be written as

3 $$F_x = - \gamma V + C_{10} + C_{11}P_A + d \cdot \beta P_A\dot x$$

**Fig. 1:**
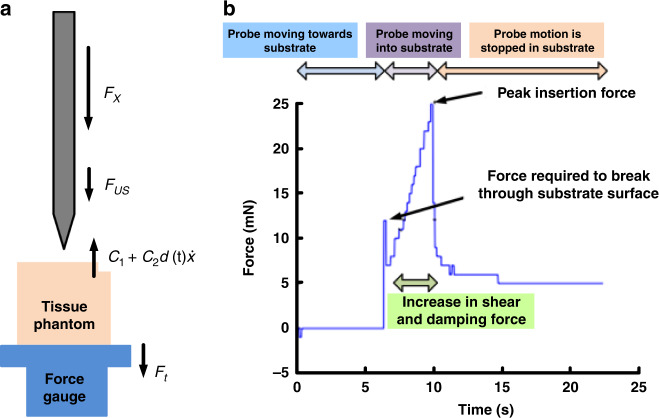
Tissue insertion force experiment and measurement. **a** The ultrasonically actuated probe was inserted into an agar tissue phantom and placed on a load cell. **b** A typical load cell force measured during insertion

In the experiments described here, the probe was inserted into the tissue at different velocities *x*˙ and stopped at a fixed depth while being driven at different drive voltages. The final peak force can then be written as

4 $$\begin{array}{l}F_x = - \gamma V + C_{10} + C_{11}P_A + d_{final} \cdot \beta P_A\dot x\\\quad\;\, = - \gamma V + C_{10} + C_{11}P_A + \delta P_A\dot x\end{array}$$

where the product *βd*_*final*_ has been replaced by *δ*. The constants *γ*, *C*_10_, *C*_11_, and *δ* can be extracted from the data of applied force versus actuator voltage into agar tissue simulants with varying agar concentration *P*_*A*_. The total force, *F*_*x*_, required for probe insertion in agar tissue phantoms was measured by a load cell (Fig. 1a), with *F*_*t*_ being the reaction force onto the load cell. A representative insertion force versus time measured by the load cell is shown in Fig. 1b.

During the insertion tests, the probe was first in contact with the agar tissue simulant using a motion stage. The contact force results in a high force on the sample and is then followed by puncturing the surface, resulting in a reduced penetration force. As the probe was inserted further into the agar, the shear frictional forces on the probe surfaces caused the measured force to increase. Probe motion was then reduced to zero velocity as the probe was held in the tissue phantom. The tissue then quickly relaxed around the probe, and the force was reduced to a residual value greater than before the insertion but much less than required during active insertion. This residual force was likely due to residual shear stress around the probe. Peak insertion forces with various values of agar gel substrate percentage (*P*_*A*_), which influence the tissue stiffness, transducer driving voltages (*V*) and insertion speeds (*x*˙), were measured and fitted using a nonlinear regression *χ*-squared minimization function. We obtain

5 $$\begin{array}{l}F_{peak} = - \left( {0.30 \pm 0.18} \right)V - \left( {3.93 \pm 0.97} \right) \\\qquad\quad\;\, +\, \left( {5.90 \pm 0.52} \right)P_A + \left( {1.18 \pm 0.26} \right)P_A\dot x\end{array}$$

**Fig. 2:**
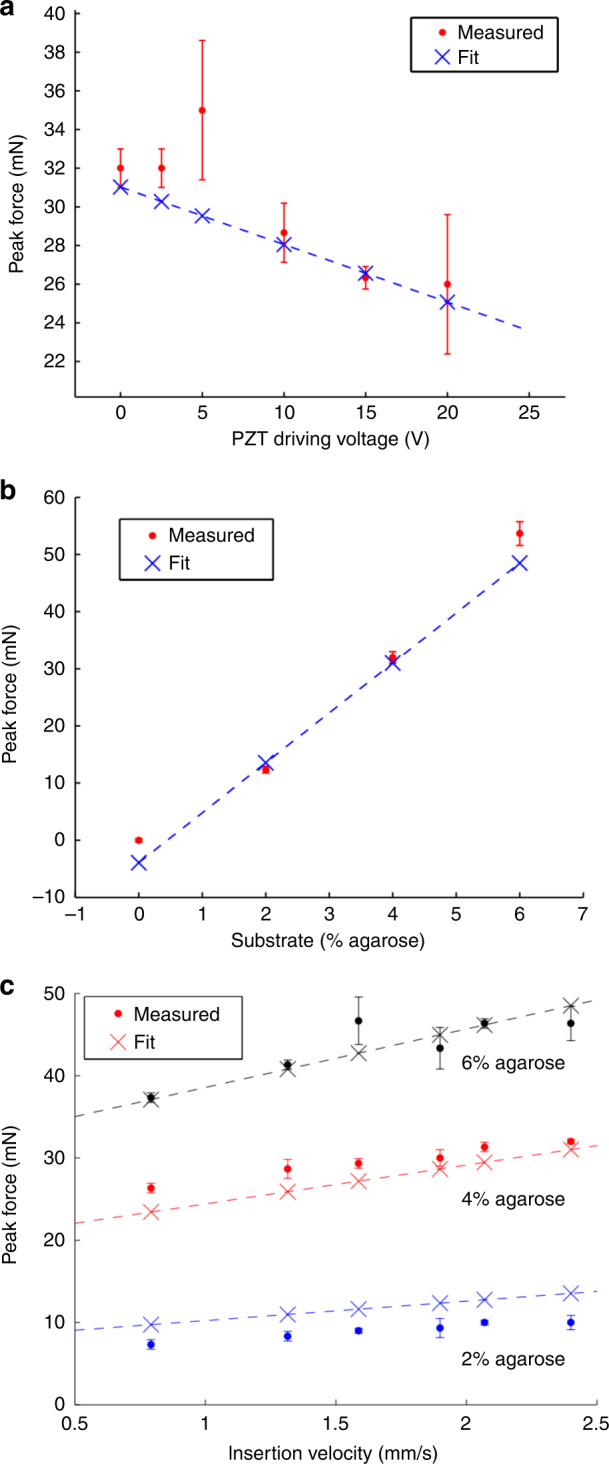
Insertion force versus PZT drive voltage, agarose concentration, and insertion velocity. **a** The force decreased with increasing PZT drive voltage for an insertion velocity of 2.4 mm/s and an agarose concentration of 4%. **b** The peak force increased with increasing agarose concentration for an insertion velocity of 2.4 mm/s and a PZT drive voltage of 0 V. **c** The peak force increased as a function of insertion velocity for different agarose concentrations and a PZT drive voltage of 0 V

The measured average peak force and the model results are shown as a function of the transducer drive voltage in Fig. 2a, the percentage of agarose in the gel in Fig. 2b, and the insertion velocity in Fig. 2c. The data indicate that by reducing the insertion velocity and maximizing the transducer driving voltage, the forces felt by the agar gel can be minimized using ultrasonic actuation of the neural probe.

The model presented in Eqs. (1) to (5) represents a linear model of forces, including a linear viscoelastic force. The tissue viscoelastic losses can be modeled using a number of models. These include the Kelvin–Voigt model, Maxwell model, generalized Maxwell model, Zener model, and the Kelvin–Voigt fractional derivative model^36,37^, which increase due to nonlinear viscoelastic coefficients. Each of the models has a specific frequency response, and the data presented in this paper can be used to confirm these models. However, the experiments were carried out at one frequency, owing to the resonance frequency of the device.

### Probe temperature and vibration amplitude during probe actuation in the air

One of the effects of the ultrasonically driven neural probes can be the heating of tissue and probe due to frictional dissipative forces between the probe and tissue interface. Additional heat sources, such as the high loss-tangent shear stress in the adhesive between the PZT plate and the silicon horn, can heat the silicon microprobe. The elevated temperatures can induce greater diffusion of biochemicals in the tissue and even induce thermal damage at sufficiently high temperatures. To assess the extent of thermal heating of the probes, an IR camera (FLIR, model T300) was used to measure the surface temperature of the probes in air while being driven at different amplitudes for different periods of time.

**Fig. 3:**
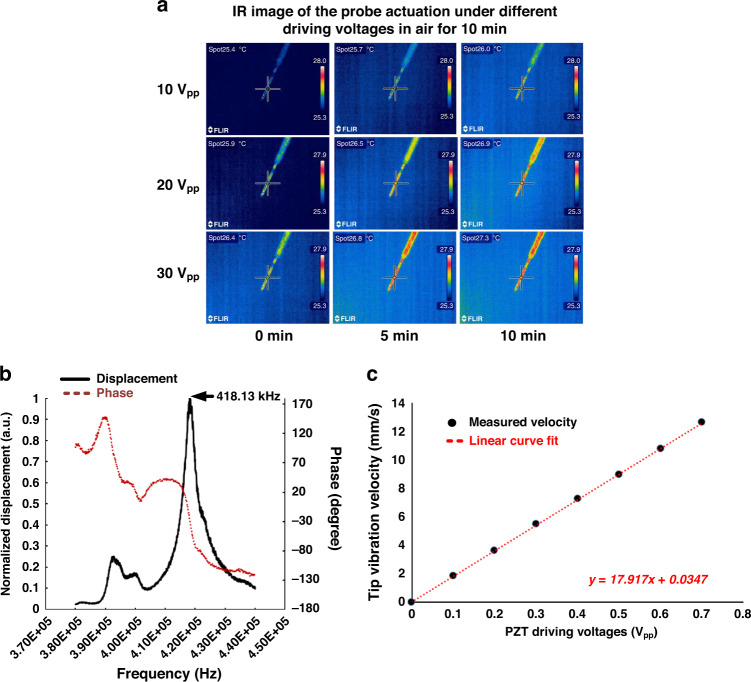
Ultrasonic probe heat generation and displacement characterization. **a** The temperature of the probe surface measured using an IR camera. The temperature was measured in air at different ultrasonic drive amplitudes as a function of time. Maximum temperature changes of ~2° were measured over 10 min at the highest 30 V_pp_ drive voltage. **b** Interferometric measurement of the longitudinal displacement. **c** Probe tip vibration velocity versus driving voltages

Figure 3 shows the temperature rise of the microprobes in the range of a few degrees above room temperature even at high drive voltages. The data were taken with the probe operating in air, not in water or tissue, due to complications of IR measurements through the water and tissue surface. We believe the IR data in the air are a good surrogate for data in tissue or water, as the expected rise in temperature in water can be less than that in air. For heat from the tissue, the probe interface, and the transducer itself, the temperature of the probe would be related to the rate of heat loss to the surroundings, including the tissue and the mechanical anchor of the ultrasonically-driven probe. Assuming that the motion of the probe is mostly longitudinal, the motion of the probe generates shear waves in the surrounding liquid on most of its surface^38^. The shear waves exponentially decay as they pass into the liquid, heating the fluid. The heat generated by the shear waves is proportional to the product of the probe velocity and tissue kinematic viscosity *P*_*gen*_ = *νv*. Even though the bulk viscosity of liquids is generally higher than that of gases, the kinematic viscosity of liquids is lower than that of gases due to the much higher density of liquids. The kinematic viscosities of air and water at room temperature are 15.6 *×* 10^*−*6^ m^2^/s and 10^*−*6^ m^2^/s, respectively. Hence, for a given surface velocity, the rate of heat generation in the air is expected to be higher. For heat transfer in a semi-infinite media from a constant surface heat source, the temperature at the surface rises with time, which is proportional to ${{\Delta }}T\sim P_{gen}t^{1/2}/\rho C_p\kappa$. The quantity $I = \sqrt {\rho C_p\kappa }$ is the thermal inertia of the media. The thermal inertia of air and water at room temperature are 5.43 and 1585 $J_m^{ - 2}K^{ - 1}s^{ - 1/2}$, respectively. Hence, the temperature rise in water would be smaller owing to the much larger thermal inertia for equal heat generation sources. Since a temperature change of 1–2° was measured in air, much smaller changes are expected in water, leading to the conclusion that the increase in tissue temperature is not significant at the ultrasonic velocities that demonstrated efficacy in reduced tissue damage.

The probe tip vibration amplitude is also characterized through the velocity of the probe in air as a function of applied voltage at the resonance frequency (Fig. 3b). Due to the limited range of focus of the interferometer (Polytec, OFV2700), the probes were driven by less than 1 V_pp_ sinusoid waves instead of the typical 10–30 V_pp_ used during neural-probe insertion. The frequency sweep of the ultrasonic probe revealed the maximum displacement at a frequency of 418.13 kHz, which closely matches the longitudinal resonance of the ultrasonic probe (408.95 kHz) given by the finite element simulations. Small variations in probe longitudinal resonance are expected to arise from the fabrication tolerance and bonding alignment. The measured values of oscillation amplitude under different driving voltages and fits are shown in Fig. 3c. Assuming an approximately linear PZT drive and tip displacement relation that will hold given a general forced response to a sinusoidal input driving function, the tip displacements for the probe with driving voltage can be estimated as

6 $${{{\mathrm{Amplitude}}}}\left( {{{{\mathrm{nm}}}}} \right) = 7.11 \times Drive\;voltage\left( V \right) + 0.0234$$

From Eq. (6), the estimated tip displacement amplitudes at driving voltages of 10, 20, and 30 V_pp_ are 71.12 nm, 142.22 nm, and 213.32 nm, respectively, in air. Using the expression $v = 2\pi fu_0$, (where *v* is the tip vibration velocity, *f* is the frequency and *u*_0_ is the tip displacement), the tip vibration velocity can be calculated as 182.74 mm/s, 365.44 mm/s, and 548.13 mm/s, respectively, which are two orders of magnitude higher than the insertion feeder velocity of 2 mm/s.

### Insertion force, wound properties, and acute inflammation in the mouse brain

**Fig. 4:**
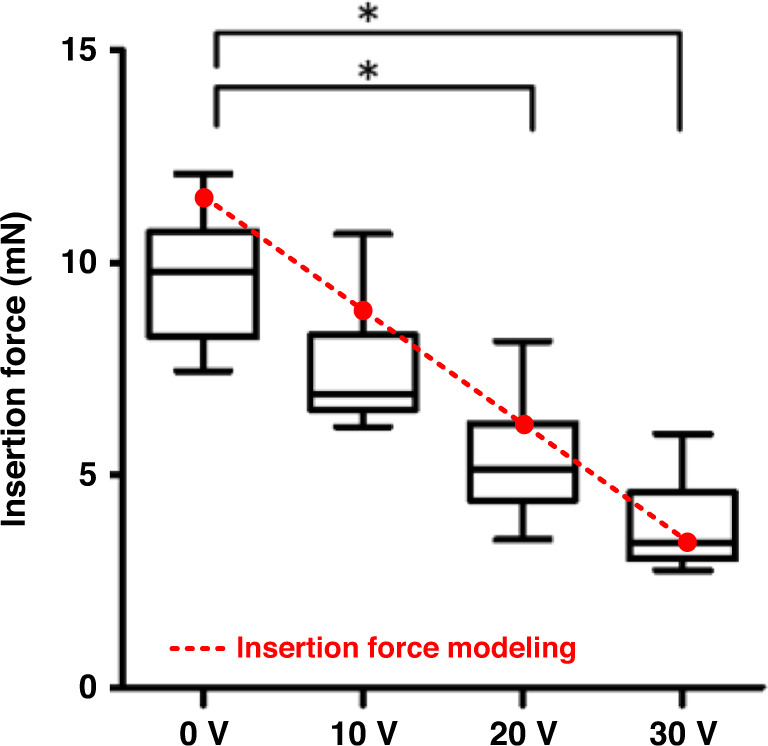
Insertion force v.s. piezoelectric driving voltages. Plot of the insertion force required to advance a probe at 2 mm/s into live mouse brain for different PZT driving voltages and the predicted insertion force based on Eq. (5) (*P < 0.05)

A strong reduction in the force required to advance the probe into the cortex of live, anesthetized mice at 2 mm/s with increasing ultrasonic drive voltage (Fig. 4; 160 insertions across 16 mice) was measured. The statistics used for the data analysis were one-way ANOVA with Tukey’s post hoc test.

In addition, the residual stress in the tissue, owing to tissue stiction and tissue compression, evident from the residual force on the probe after the insertion was completed, was generally reduced with ultrasonic actuation, as shown in the part of Fig. 1. The reduction in residual force was not measured extensively and hence is only presented here as a point of interest for future studies.

**Fig. 5:**
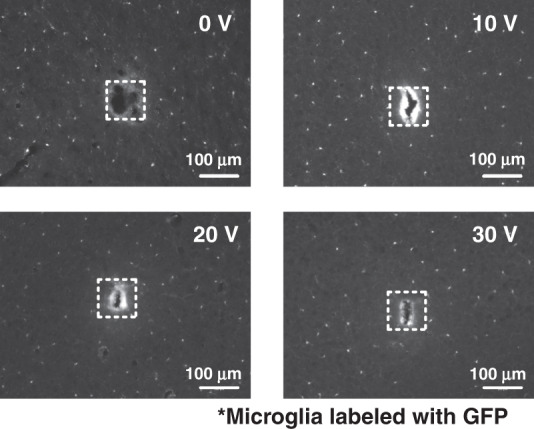
Fluorescence images of brain sections cut parallel to the cortical surface showing insertion sites ~1 h after inserting and removing the probe with different ultrasonic driving voltages. The white dotted square represents the probe tip size. The visible cells are microglia labeled with GFP

**Fig. 6:**
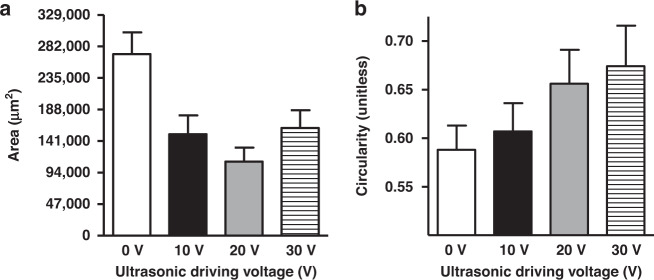
Wound properties of the insertion site. **a** Area and **b** circularity of wound left by insertion and removal of the probe as a function of ultrasonic drive voltage. The number of insertions: 34 insertions at 0 V, 23 insertions at 10 V, 19 insertions at 20 V, and 13 insertions at 30 V

The probe was removed approximately one minute after insertion at the same velocity as the insertion velocity. After the animal was sacrificed, each insertion site was extracted in 30-µm thick slices cut parallel to, and at a depth of ~300 µm beneath, the cortical surface (Fig. 5). The cortical slices were imaged using an optical microscope, and three metrics were quantified to characterize the degree of tissue injury associated with electrode insertion. First, we characterized the wound size, with larger wounds indicating greater damage. Second, we measured the perimeter to area ratio of the wound, which increases with irregularity of the wound border, which we interpret as indicating greater tissue tearing and injury. Third, we quantified the microglial density near the wound as a measure of the intensity of the acute inflammatory response. We found that ultrasonic actuation led to the decreased area (Fig. 6a) and increased circularity (Fig. 6b) of the wound left by probe insertion and retraction, with circularity is defined as:

7 $$f_{circ} = \frac{{4\pi A}}{{P^2}}$$

where *A* is the area and P is the perimeter of the wound left by probe insertion. A circularity of *f*_*circ*_ = 1 would indicate a perfect circle, while smaller values would indicate an increased perimeter corresponding to roughness along the wound border. Insertion without any ultrasonic drive (0 V drive) resulted in a larger wound area with a more irregular perimeter, likely indicative of tissue tearing during insertion, compared to the smaller wounds with smoother perimeters seen when inserted with ultrasonic actuation.

**Fig. 7:**
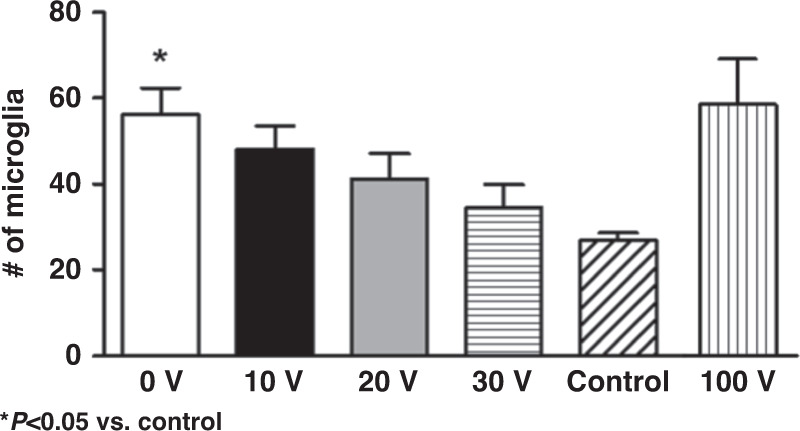
Number of microglia within 200 × 200 µm of the probe insertion site in a 30-µm thick section cut perpendicular to the probe insertion direction under different driving voltages during probe insertion. Control regions had no nearby probe insertion (*P < 0.05, one-way ANOVA with a Holm‒Sidak correction for multiple comparisons)

The acute inflammatory response was evaluated by quantifying the number of microglia, which expressed GFP in these mice, within a 200 µm area around the electrode path for these same tissue sections. The microglial density near the wound is expected to increase in the 1 h between electrode insertion (and removal) and euthanasia of the animal due to migration of microglia toward the injury^39^. We observed increased tissue autofluorescence at the borders of the wound, which could not be spectrally separated from the GFP fluorescence from microglia. We found a trend suggesting a decreased number of microglia with increased actuation voltage compared to control regions far from an insertion site (Fig. 7). However, we also discovered confounding effects with extremely high actuation voltages (100 V); the microglial cell counts increased, indicating that the benefit of ultrasonic insertion was reversed.

Taken together, these data suggest that ultrasonic actuation during electrode insertion reduces the initial tissue damage and acute microglial activation to near-baseline levels. This reduction in injury could increase neural microelectrode performance.

### Chronic inflammation and recording in mouse brain

**Fig. 8:**
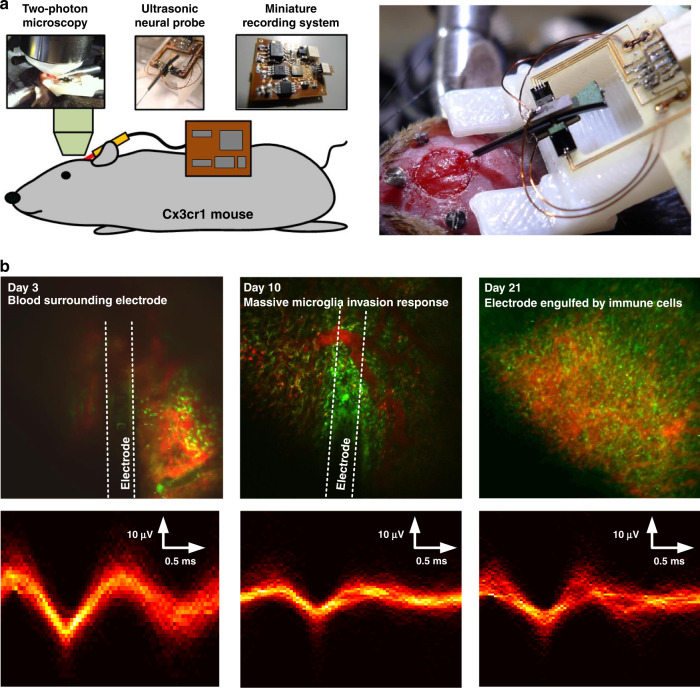
Chronic experiment setup and measurement. **a** Chronic animal model with a cranial window for two-photon excited fluorescence imaging at the tip of the inserted ultrasonic neural probe and the miniature recording system (left). Picture of the implanted ultrasonic neural probe (right). **b** Two-photon imaging and electrical recording over time after electrode implantation. In the images at the top, the green fluorescence is from GFP-labeled microglia, and red fluorescence is from the vasculature, labeled by intravenous injection of Texas Red-dextran. On day 3, the two-photon image is blurry due to hemorrhaging from the implantation. On day 10, increased microglial cell density was observed at the implantation site. By day 21, the recording electrode was engulfed by microglial cells. The traces at the bottom show the overlaid extracellular recording traces from individual neurons that have been separated using spike sorting

**Fig. 9:**
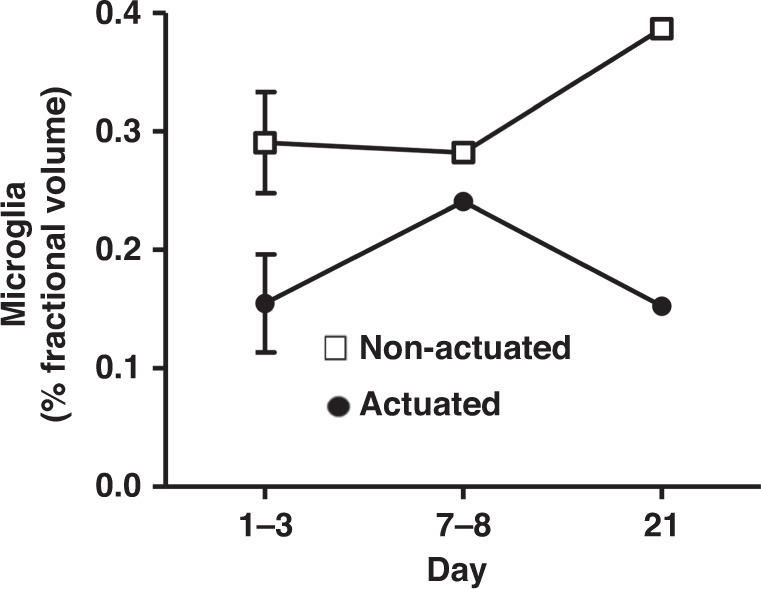
The fractional volume of the tissue taken up by microglia near the electrode over time. Over time, ultrasonic actuated insertion led to a smaller increase in microglia than nonactuated insertion

To evaluate if ultrasonic actuation decreases chronic inflammation and increases the fidelity of neural recordings, we implanted probes in 13 mice, 7 with ultrasound actuation at 20 V and 6 without. We used two-photon excited fluorescence microscopy to image microglia (which expressed GFP) near the probe, and we recorded activity from single neurons in the days and weeks after electrode implantation (Fig. 8). An inflammatory response, indicated by an increase in microglial density, was elicited in both groups due to the insertion trauma inflicted on the surrounding tissue. The electrode was typically engulfed by microglia by day 10, with the microglia density greatest within 25 µm of the electrode. The density of microglia, as measured by the volume fraction of the tissue that was GFP-positive, continually increased over the 21 days of observation in the nonactuated group but increased less and appeared to level off and even decrease after one week in the actuated group (Fig. 9). Due to the electrode puncturing the surrounding vasculature upon insertion, leakage of plasma around the electrode was typical from days 1–3 (Fig. 8), and in some instances, evidence of microhemorrhaging was seen beyond this time frame. These results suggest that ultrasonic actuation of the probe reduced the inflammatory response shortly after the injury, consistent with our acute studies (Fig. 7), and that this reduction in inflammation persists over time (Figs. 8 and 9).

**Fig. 10:**
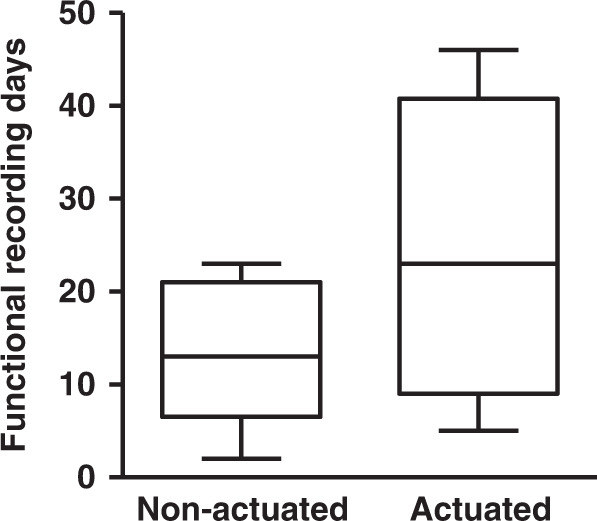
Functional recording days v.s. insertion methods. Length of time, in days, that high-quality extracellular recordings of individual neurons could be achieved for electrodes inserted with and without ultrasonic actuation

We performed electrophysiological recordings with the inserted electrode, used spike sorting to isolate the signal from individual neurons, and determined the SNR of the electrical recordings we were able to acquire. We then characterized how long after probe implantation we could achieve functional recordings, defined as an SNR > 2 and no catastrophic failure (e.g., electrode breakage). We found a trend toward longer recording capabilities in the ultrasound-actuated group than in the nonactuated group (Fig. 10).

## Discussion

The longevity of electrical neural interfaces is critical to achieving brain–machine interfaces that can be translated into treating diseases in humans and not just used for laboratory research in animals. One of the challenges to realizing long-term reliable neural interfaces is the inflammatory response to the inserted probes. This inflammatory response can cause the electrical interfaces to become progressively ineffective by a buildup of an insulating coating on the probe electrode and can lead to progressive damage to nearby neurons. This study explored the use of ultrasonic actuation during probe insertion to reduce this inflammatory response. We demonstrated a process to fabricate an integrated ultrasonic transducer with neural recording electrodes and strain gauges. Using these probes, we found that ultrasonic actuation leads to a reduced insertion force in tissue membranes and in the mouse brain. These findings are in broad agreement with earlier work in other tissue types that has shown that ultrasonic actuation reduces the insertion force required to penetrate tissue. In mice, we further found a smaller and more regularly shaped wound, decreased acute and long-term activation of brain microglia, and a longer duration of high-quality single neuron recordings for electrodes inserted with ultrasonic actuation.

A major limitation of the current ultrasonically actuated probe we have built is that the probe itself is an integral part of the transducer driver. This leaves a relatively large probe attached to the mouse, increasing the risk of mechanical disruption to the probe. A probe that could enable the ultrasound transducer to be disconnected from the inserted neural probe once it was in place could mitigate this problem. We have recently conducted preliminary work on such a design^40^, where the silicon horn driver is disconnected by dissolving a water-soluble adhesive bond between the neural probe and the ultrasound driver.

## Methods

### Probe design methodology

**Fig. 11:**
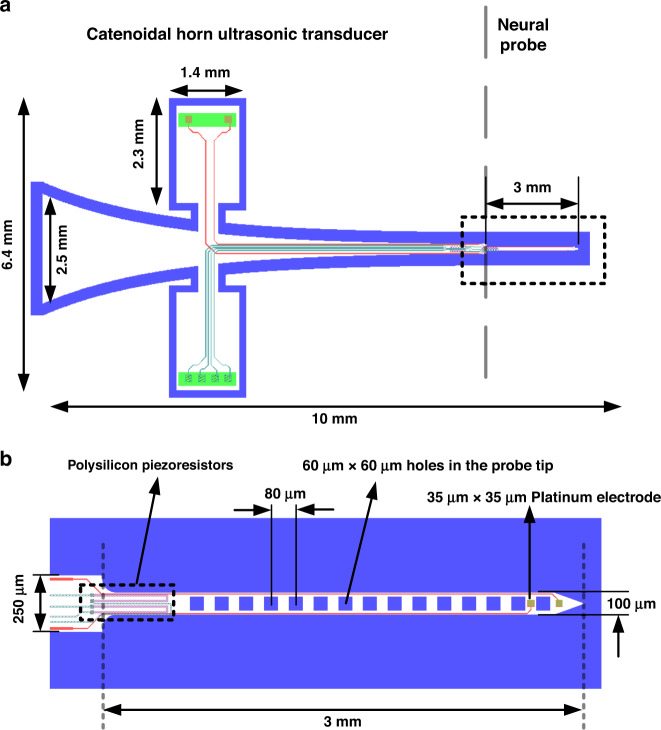
Ultrasonic transducer design of the integrated neural probe. **a** Catenoidal horn and co-fabricated neural probe and **b** integrated polysilicon piezoresistors and platinum recording electrodes

Ultrasonic horn transducers are ultrasonic wave resonators with a tapered cross-sectional area to concentrate mechanical energy at the tip of the transducer with a small sectional area, thereby achieving high vibration amplitudes^41^. Several horn shapes have been previously investigated, including exponential, linear, stepped, and catenoidal horns^33,42,43^, with each horn type having its own relationship between the cross-sectional area and the horn axis. Each horn shape offers tradeoffs in displacement magnification versus maximum stress in the horn. A catenoidal horn shape allows for a large displacement amplification between the shank and tip of the probe while minimizing the stress concentration^33^. Silicon ultrasonic horn-based transistors, driven by bulk piezoelectric plates, have been emerging as suitable transducers due to their superior material properties and capability for the integration of microfabricated sensors. Strain and bioelectrical potential measurement sensors can be integrated for closed-loop control of transducers in applications such as phacoemulsification microsurgery^44^, testicular tubule assay^45^, blood viscosity sensing^46^, and ventricular fibrillation monitoring^47^. To enable ultrasonic vibration of the neural-probe tip during probe insertion into tissue, a silicon-based neural probe is co-fabricated with a catenoidal ultrasonic horn, as shown in Fig. 11.

The cross-sectional area of a catenoidal horn is defined by

8 $$A\left( x \right) = A_1 \cdot \cosh ^2(\alpha \left( {L - x} \right))$$

where

9 $$\alpha = \cosh ^{ - 1}(\sqrt {A_0/A_1} )/L$$

where *A*_0_ and *A*_1_ are the cross-sectional areas of the probe at the tip, and the shank end, respectively, and *L* is the length of the horn. A neural-probe prong with a width of 100 µm is formed at the tip of the main horn. The mass and length of the neural-probe prongs are much smaller than the mass and length of the ultrasonic horn and thus do not significantly affect the ultrasonic resonance mode displacement of the horn. The prong includes two platinum electrical recording sites (35 × 35 µm) and integrated strain gauges at the interface of the horn and the prong to measure the strain as the probe is inserted into the tissue. The strain gauges consist of polysilicon resistors, whose resistance changes as the probe bends and compresses, allowing monitoring of the strain on the probe. Holes are included in the neural probes (60 × 60 µm, with 80-µm pitch) to enable tissue to grow through the probe for better tissue adherence, as suggested by previous work on sieve electrodes for nerve regeneration studies and peripheral nerve recording, which demonstrated improved electrical contact^48^.

### Probe-fabrication procedure

**Fig. 12:**
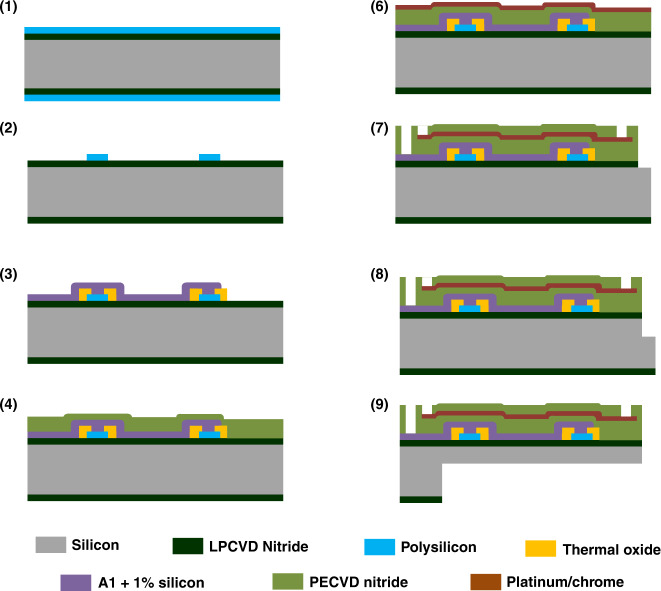
Fabrication process flow. Fabrication process flows for the ultrasonic neural probe with integrated strain gauges

The fabrication process flow has been previously developed^47^ and was modified for ultrasonic-enabled neural probes, as shown in Fig. 12.

The process starts with a four-inch diameter <100> silicon wafer coated with a 600-nm thick film of low-pressure chemical vapor deposition (LPCVD) silicon nitride, followed by a 600-nm thick LPCVD undoped polysilicon film on both sides of the wafer (Fig. 12(1)). The silicon nitride insulation layer serves to isolate the metal signal lines from the silicon substrate and impedes the formation of Schottky electrical contacts to silicon. The polysilicon film was ion-implanted with boron at a dose of 2 × 10^15 ^ions/cm^2^ at 100 keV with a 7° tilt angle to form a piezoresistive layer. After annealing in nitrogen at 950 °C for 60 min, the measured sheet resistance of the piezoresistive film was 180 Ω*/*Q to 190 Ω*/*Q. The piezoresistive polysilicon film was then patterned to form piezoresistors with a resistance of 13 kΩ (Fig. 12(2)). A 300-nm thick film of thermal oxide was grown on top of the piezoresistor as an insulation layer and patterned to expose the piezoresistor to sputtered aluminum alloy (aluminum + 1% silicon) metal lines (Fig. 12(3)). The wafers were then annealed at 250 °C with 5% hydrogen and nitrogen again to activate the dopants and produce good electrical contact between the aluminum and piezoresistors. Insulating plasma-enhanced chemical vapor deposition (PECVD) low-stress nitride was deposited at 250 °C to ensure thermal compatibility with the low melting temperature metal on the wafer (Fig. 12(4)), followed by platinum evaporation to define electrical recording sites (Fig. 12(5)). Platinum was selected as the metal interface to the electrolyte due to its excellent biocompatibility and chemical stability. To promote the adhesion of platinum to silicon nitride, a 25-nm layer of chrome was evaporated first as an adhesion layer followed by 250 nm of platinum. Another insulating PECVD low-stress nitride was deposited and patterned to define the electrical recording sites and the bond pad area (Fig. 12(6)). Two steps of deep silicon reactive ion etch (DRIE), front-side (Fig. 12(7)) and back-side (Fig. 12(8)), were performed to release the shape of the structure. The probe tip thickness, 100 µm, was defined by a front-side DRIE etch. For back-side etching, a polymer coating of Protek SR-25 was spun to protect the front-side features during DRIE probe release.

### Probe assembly procedure

**Fig. 13:**
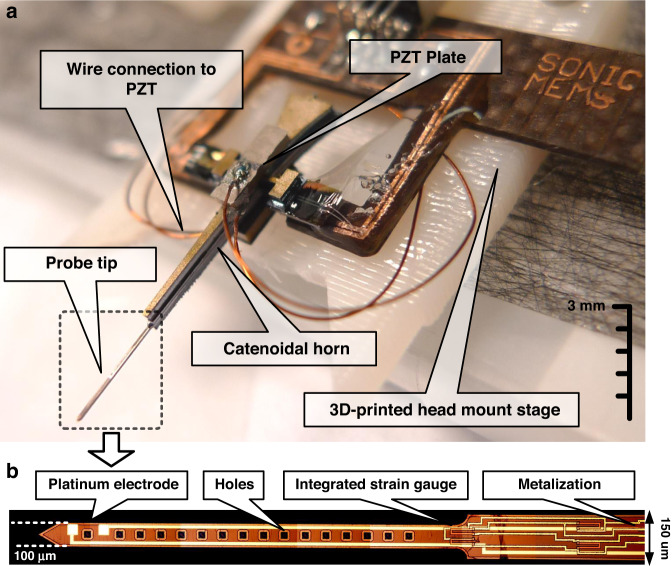
Picture of the assembled probe device. **a** Miniature silicon horn with piezoelectric plates to drive the probe at its longitudinal resonance. **b** Micrograph of the probe tip with integrated strain gauges and platinum recording sites

A dummy horn structure without the prong was adhesively bonded to the horn with the prong to balance the transverse motion during ultrasonic actuation. Two PZT piezoelectric plates (3.55 × 1.25 × 0.5 mm) were adhesively affixed at the location of the zero-displacement nodes of the longitudinal mode. The PZT was actuated at its ${\frac{ {{\lambda }}}{ 2 }}$ resonance, which matched the longitudinal resonance of the silicon probe to maximize the energy coupling from the PZT plates to the silicon structure^49^. The entire structure was then placed on a custom-printed circuit board (PCB) with a 3D-printed head stage for chronic animal implantation. A picture of the final assembled device is shown in Fig. 13.

### Tissue-simulant preparation and load cell setup

A procedure was developed for measurement of the insertion force with and without ultrasound in agarose gel, which serves as a tissue simulant. Agarose (Agar Powder, Acros Organics) gels of various concentrations (weight/volume (w/v)) were prepared by dissolving powdered agarose in distilled water. The solution was sealed and heated for 20 min at 90–95 °C on a hot plate and stirred frequently by hand until dissolved completely. The solution was then poured into Petri dishes to cool at room temperature and cure for at least 6 h. A glass slide was placed on top of the force transducer (Chatillon DGGS) and the agarose gel was placed on top of the glass slide. The ultrasonic probe was mounted on a motorized micromanipulator (MP285, Sutter Instrument). Insertion forces were measured while the probe was inserted into the tissue simulant at various constant velocities. The force transducer and micromanipulator were all affixed on an optical table to minimize the misalignment between the probe tip and tissue stimulant.

### Insertion force, wound properties, and acute inflammation in mouse brain procedures

The care and experimental manipulation of animals used in this study were approved by the Institutional Animal Care and Use Committee at Cornell University. The mice used in this study had one copy of the CX3CR1 fractalkine receptor gene replaced with the gene for green fluorescent protein (GFP), leading to GFP expression in all brain microglia^50^. Animals were anesthetized with 5% isoflurane (VetOne) in 100% oxygen and maintained at 1.5–2% for the duration of the experiment. At the beginning of the surgery, mice received an intramuscular injection of glycopyrrolate, an anticholinergic, at 0.002 mg/100 g mouse weight to assist in keeping the airways clear of fluid buildup. Mice were then secured in a stereotaxic frame using standard ear bars, and bupivacaine (0.125%, 0.1 ml) was injected subcutaneously above the skull. Opthalmaic ointment was applied to both eyes to prevent drying. A short incision was made on top of the skull, and the connective tissue was scraped away, exposing the bone. A 5-mm diameter opening was drilled in the skull, and the dura mater was left intact. The ultrasonic neural probe was mounted at an angle of 90° relative to the cortical surface on a motorized micromanipulator (World Precision Instruments, SM325). The probe was then inserted at a speed of 2 mm/s to a depth of approximately 6 mm. During the insertion, integrated strain gauges were used to measure the insertion force. The probe was then removed ~1 min after insertion. Approximately 10 such insertions were made in each animal at ultrasound drive voltages of 0, 10, 20, and 30 V. The drive voltages were randomized across the insertion locations. Approximately 45 min after the last insertion, the animal was sacrificed, and the acute inflammatory response and degree of tissue injury were assessed histologically. Briefly, mice were transcardially perfused with phosphate-buffered saline (PBS, pH 7.4, Sigma Aldrich) followed by 4% paraformaldehyde (PFA) (Thermo Fisher Scientific) in PBS. Following perfusion, brains were removed and immersed in 30% (w/v) sucrose until saturated. For sectioning, brains were frozen in optimal cutting temperature (OCT) compound (Tissue-Tek), and 30-µm thick sections were cut parallel to the cortical surface on a cryotome (Microm HM550, Thermo Fisher Scientific). Images of the brain sections were captured with an Olympus BX41 wide-field fluorescence microscope using standard filter sets for GFP to visualize microglia.

### Chronic animal preparation with probe and window

Preoperative care was administered using aseptic techniques for chronic animals, and instruments and materials were sterilized by autoclaving. Animals were anesthetized and prepared for surgery as described above. Ketoprofen (5 mg/kg) and dexamethasone sodium phosphate (0.2 mg/kg) were administered subcutaneously for pain management and to minimize edema, respectively. After the skull was exposed and connective tissue was removed, the dry skull was coated with a thin layer of cyanoacrylate adhesive (Vetbond). Three screws were placed in the skull to provide a framework for the dental cement to create a strong adhesion. A 5-mm diameter craniotomy was then performed. The probe tip was carefully lowered to the caudal edge of the craniotomy using a precision motorized micromanipulator and oriented at an angle of 10° relative to the cortical surface. The probe was inserted at a speed of ~0.5 mm/s into the brain until the probe tip was ~200 µm under the surface of the cortex and centered under the craniotomy (Fig. 8a). The probe was actuated at 0 or 20 V during insertion. After insertion, the probe was secured to the base of the skull with dental cement. A glass coverslip was then glued to the bone over the craniotomy site with cyanoacrylate and dental cement. The space under the window was filled with artificial cerebral spinal fluid (ACSF). Finally, the skin was closed around the margins of the cranial window with cyanoacrylate.

### In vivo imaging and quantification of microglial responses

To track the microglial response to electrode insertion, we used the mice expressing GFP in brain microglia. The inflammatory response to the inserted electrode was visualized using a custom-built two-photon excited fluorescence (2PEF) microscope. Before imaging sessions, mice were anesthetized with isoflurane (1.5–2%) and retro-orbitally injected with Texas Red-dextran (0.05 mL of 2.5% w/v; D1830; Invitrogen, Carlsbad, CA, USA) in physiological saline to label the blood plasma. Animals were transferred to the microscope, and anesthesia was maintained at 1–2% during the duration of the imaging session. Opthalmaic ointment was applied to both eyes. Two-photon excitation used 800-nm light from a Ti:Sapphire laser (Mira-HP, Coherent, Inc.). For visualization of GFP (microglia) and Texas Red-dextran (vasculature), the fluorescence emission was split with a 605-nm longpass dichroic and relayed through bandpass filters of 575/25 and 645/65 nm (center wavelength/bandwidth), respectively. Laser scanning and data acquisition were controlled by Scan-Image (Vidrio, Inc.). All 2PEF in vivo images were analyzed using ImageJ (NIH). All images were first filtered using a one pixel radius median filter. The density of microglia next to the interface of the electrode was evaluated by finding the fractional volume that was GFP-positive for three time periods: 1–3, 7–8, and 21 days after electrode implantation.

### In vivo recording and quantification of neural activity

Neural activity was recorded from the inserted probes in ambulatory mice. The recording used an AC amplifier at a gain of 100 (A-M system, Model 1800) and was acquired with a data acquisition system at a sampling rate of 40 Mbps (National Instrument, USB-6259 BNC) for 30 min for each mouse during each recording session. Spikes from individual neurons were then sorted using UltraMegaSort 2000^46,47^. The signal-to-noise ratio (SNR) for each neuron was then defined as the average action potential peak-to-peak height (APheight) divided by two times the standard deviation of the mean background noise level (STDnoise) over the 0.3 ms preceding the spike^48^.
